# Assessment and parameter identification of simplified models to describe the kinetics of semi-continuous biomethane production from anaerobic digestion of green and food waste

**DOI:** 10.1007/s00449-016-1577-x

**Published:** 2016-03-09

**Authors:** Raymond O. Owhondah, Mark Walker, Lin Ma, Bill Nimmo, Derek B. Ingham, Davide Poggio, Mohamed Pourkashanian

**Affiliations:** Energy Research Institute, School of Chemical and Process Engineering, University of Leeds, Leeds, LS2 9JT UK; Energy Engineering Group, Department of Mechanical Engineering, University of Sheffield, Sheffield, S10 2TN UK

**Keywords:** Anaerobic digestion (AD), Modelling, Food waste (FW), Green waste (GW), Parameter estimation, Parameter identification

## Abstract

Biochemical reactions occurring during anaerobic digestion have been modelled using reaction kinetic equations such as first-order, Contois and Monod which are then combined to form mechanistic models. This work considers models which include between one and three biochemical reactions to investigate if the choice of the reaction rate equation, complexity of the model structure as well as the inclusion of inhibition plays a key role in the ability of the model to describe the methane production from the semi-continuous anaerobic digestion of green waste (GW) and food waste (FW). A parameter estimation method was used to investigate the most important phenomena influencing the biogas production process. Experimental data were used to numerically estimate the model parameters and the quality of fit was quantified. Results obtained reveal that the model structure (i.e. number of reactions, inhibition) has a much stronger influence on the quality of fit compared with the choice of kinetic rate equations. In the case of GW there was only a marginal improvement when moving from a one to two reaction model, and none with inclusion of inhibition or three reactions. However, the behaviour of FW digestion was more complex and required either a two or three reaction model with inhibition functions for both ammonia and volatile fatty acids. Parameter values for the best fitting models are given for use by other authors.

## Introduction

The increase in the urban population worldwide has led to an increase in urban solid waste generation. The conventional method of waste disposal by landfilling is not favourable and no longer viable in many places due to lack of suitable sites, fugitive methane emissions, and groundwater pollution, which has led to strong legislation in some countries. Anaerobic digestion (AD) technology offers an alternative disposal route for organic waste with several inherent advantages, such as energy production through biogas (methane and carbon dioxide) and the production of nutrient rich liquid by-products that can replace synthetic fertilisers. This has led to the development of various reactor designs, as well as research into optimal operating conditions and the microorganisms involved [1].

AD is a complex degradation process consisting of a diverse population of microorganisms converting a wide range of long chain organic molecules into simpler compounds, eventually resulting in complete conversion of the degradable carbonaceous material into methane and carbon dioxide. From a mathematical point of view the system is inherently non-linear in nature and easily influenced by changes in the process parameters and operating environment. To better understand the process for the purpose of design, optimisation and control, the IWA developed the anaerobic digestion model no. 1 (ADM1) [2] which contains 26 dynamic states, including nine microbial populations catalysing 12 biochemical reactions, the ionic balance governing pH and the liquid–gas transfer process. The complexity of the model contributes to its major setback as it makes the identification of parameters very difficult, thus leading to structural weaknesses in the model [3, 4]. The application of ADM1 model sometimes involves the modification of the model structure for different types of feedstock and to extend the model to processes that were not included when the model was developed [3].

Over the years, several simplified models of the AD process have being proposed with the aim of reducing the complexity in terms of the number of parameters to be identified and also for specific problems, including the development of a framework for monitoring and controlling [5]. Other applications of these simplified models include; the optimization of methane production [6], the dynamic modelling of the behaviour of AD processes such as the comparisons of different reactor combinations [7], the simulation of dynamic behaviour of a two stage AD process [8] and the AD of microalgae [9]. The models can be classified in three ways; by the number of fractions that describe the complex organic matter, by the number of populations of microorganisms that catalyse the reactions, or by the number of biochemical reactions taking place. These models cannot describe many of the more complex interactions and process occurring during AD such as the effect of moisture content, application of leachate recirculation, mixing intensity, aeration, gas mixing, foaming, changes in feedstock characteristics (physical and chemical), effect of micronutrients and shifts in the populations of the microorganisms.

The simplest models involve a single population of microorganisms and one biochemical reaction where the inlet organic matter, described by a single state variable is converted directly to methane [10, 11]. The shortfall of these simple dynamic models is that they can only, at best, capture the most basic kinetic behaviours exhibited by an AD system. However, promising results can come from slightly more complex models involving two biochemical reactions that represent fermentation and methanogenesis [5–7]. Three reaction models consist of hydrolysis, acidogenesis and methanogenesis [8, 12] or consist of two hydrolysis stage reactions followed by the methanogenesis reaction [9]. However, it should be noted that in almost all cases, these models have been applied to wastewater treatment plant and liquid substrate from various industrial processes rather than solid waste as in this paper.

Going forward, this study is an assessment of the simplified AD models in their ability to reproduce the kinetics of methane production from the digestion of solid waste. Model structures similar to those proposed by Bernard et al. [5] and Mairet et al. [9] were used to assess the use of alternative kinetic equations to describe reactions rates. We have used parameter estimation as a tool to fit the models to a rich experimental dataset, using a number of model structures, combinations of kinetic equations and simple inhibition descriptions. The closeness of the fit can be used to give an insight into the important phenomena exhibited by the AD system as well as to assess the simplest model required to satisfactorily describe the digestion kinetics of complex solid wastes, such as food waste (FW) and green waste (GW).

Moreover, the kinetic descriptions used in the previous studies have been mainly implemented as either Contois or first-order for the hydrolysis step and Monod and/or Haldane for the acidogenesis, acetogenesis and methanogenesis steps of the AD process [5, 7]. However, other reaction kinetic equations show promise in replicating the observed process kinetics and the comparison of different kinetic models using linear and non-linear regression techniques to fit the experimental date from a USAB digester have been explored [13]. Hence, in the present study, a variety of kinetic combinations is considered including, the less commonly used Moser and Tessier kinetics [14].

## Methodology

### Experimental method

Segregated household GW and FW were collected at a local recycle centre (Todmorden, UK) and stored in the laboratory at a temperature of 5 °C. Within 24 h, the samples were examined and large pieces of bone, plastic, metal, wood were removed to avoid damage to the homogenisation equipment and reduce sampling errors during later analysis. The samples were then homogenised using a commercial food mincer and sampled for physio-chemical analysis. The remainder of the biomass samples was stored in a freezer with a temperature of around −18 °C and thawed before feeding to the digesters.

The semi-continuous study into the production of biogas from GW and FW was performed in two 2-l laboratory digesters. The temperature of the digester was maintained at 37 °C by immersion in a water bath and mixing was provided by a vertical stirrer operating at 60 RPM for 30 s every minute. The inoculum for the experiment was obtained from a homogenised sample of laboratory digestate from other digestion experiments, which originated from a mesophilic digester treating primary and secondary sludge at a wastewater treatment plant.

**Fig. 1:**
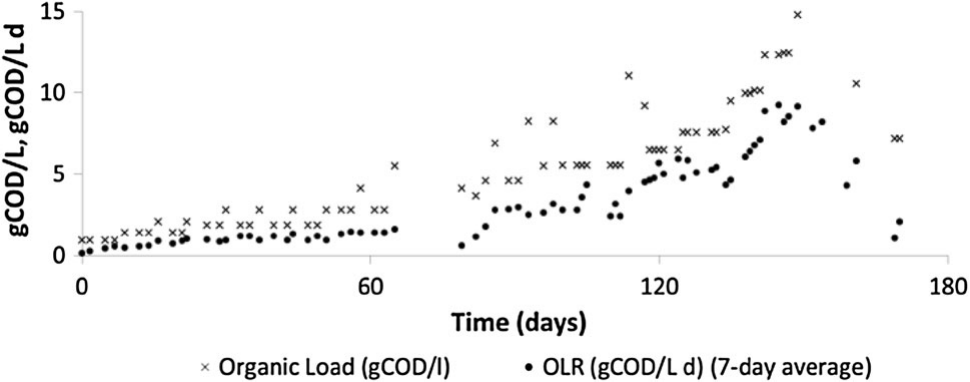
Organic loadings to the 2–l experimental digesters for both GW and FW

The two digesters were fed chemical oxygen demand (COD) equivalent pulses of GW and FW over a period of 112 and 176 days, respectively, with a gradual increase in organic loading until failure of the process occurred, as shown in Fig. 1. In the case of GW, the experiment was terminated early due to excessive foaming in the digester. The methane production of the digesters was monitored continuously and samples for offline analyses were taken intermittently during the feeding operations.

The pulsed and irregular feeding of the experimental system is usual in AD research, especially for solid waste and in small scale digesters in rural areas [15] and it is applied in the present study. This approach was chosen for two reasons; (1) the data produced is richer in kinetic information when compared with steady organic loading rate (OLR) experiments, and (2) the feeding profile is more representative of small scale systems which are manually operated and which was the focus of the larger research programme.

### Analytical methods

Measurement of the methane production for the laboratory digesters was performed using an AMPTSII gas flowmeter (Bioprocess Control, Lund, Sweden). In this system the produced biogas was scrubbed into a 3 M NaOH alkaline solution to remove the carbon dioxide and hydrogen sulphide, and its volume was determined using a multichannel volumetric measurement device with a resolution of 10 ml. Methane production was then calculated assuming a scrubber efficiency of 98 %, taking into account the overestimation caused from the initial flush gas content (nitrogen), subtracting the concentration of water vapour and reporting the volumes at STP (0 °C and 1 atm), as per the manufacturer’s guidelines.

The total solids (TS) and volatile solids (VS) were measured as per standard methods [16]. The concentration of volatile fatty acids (VFA) was measured using an Agilent 7890A gas chromatograph, with a DB-FFAP column of high polarity designed for the analysis of VFA columns, as per the manufacturer’s guidelines. Elemental analysis was determined using an elemental analyser (Flash EA2000, CE Instruments) equipped with a flame photometric detector (Flash EA 1112 FPD, CE Instruments). The theoretical chemical oxygen demand (COD_th_) was calculated from the empirical formula obtained from elemental analysis, considering the organic matter to be fully oxidised to carbon dioxide and water, with nitrogen being reduced to ammonia and sulphur oxidised to sulphuric acid [17].

### Model description

Three simplified models of AD have been considered in this paper. The models included a one reaction model (1R), a two reaction model (2R) and a three reaction model (3R) and were based on the work of Donoso-Bravo et al. [10], Bernard et al. [5] and Mairet et al. [9], respectively, with some minor modifications as discussed below. It should be noted that parameters of the model, unless calibrated as part of this work, were maintained as per the original citations and therefore there are some differences in units as described in the nomenclature section and appendices. In general the nomenclature was maintained as per Bernard et al. [5]. As part of the model screening in this work, hydrolysis was modelled using the first-order, Contois and Monod equations, and methanogenesis by Monod, Haldane, Tessier and Moser equations as described in Sect. “Kinetics of reaction”.

Using these simplified models to describe the complex AD process requires several assumptions;

- The AD process can be simplified by a limited number of reactions. For the study we consider only the hydrolysis stage and the methanogenesis stage with the other stages in the process being incorporated into the above reactions.
- The organic matter in the substrate can be represented by either a single lumped fraction in the case of the 1R and 2R models, or two fractions in the case of the 3R model.
- Inhibition only occurs in the methanogenic stage.
- The methane produced is immediately transferred into the gas phase without undergoing the liquid–gas transfer process, in contrast to ADM1 which calculation of the gas–liquid mass transfer rate and therefore includes both the dissolved and headspace gases as dynamic states.
- The digester is completely mixed and the biomass concentration is homogeneous.

The use of a completely mixed model (i.e. no spatial variation) is common in AD modelling even where solid substrates are fed to the system [18–20], and in which cases there will undoubtedly be stratification of the solid components within the system. We used an intermittent mixing regime for technical reasons (as recommended by the manufacturer) and observation of the experimental setup confirmed that the digester contents were sufficiently mixed at all times.

#### One reaction model (1R)

The 1R model used in this paper is a generic mass balance involving a single substrate (*S*_1_) that is converted to methane and carbon dioxide by the action of a single population of microorganisms (*X*_1_). The dynamic model is fully described in the following equations:

1 $$ \frac{{{\text{d}}X_{1} }}{{{\text{d}}t}} = r_{1} - DX_{1} $$

2 $$ \frac{{{\text{d}}S_{1} }}{{{\text{d}}t}} = k_{1} r_{1} + d\left( {S_{{1,{\text{in}}}} - S_{1} } \right) $$

Methane flowrate:

3 $$ q_{m} = k_{3} r_{1} $$

The reaction stoichiometry is given by Eq. 4.

AD reaction:

4 $$ k_{1} S \to X + k_{2} {\text{CO}}_{2} + k_{3} {\text{CH}}_{ 4} $$

#### Two reaction model (2R)

The 2R model includes a single lumped fraction of particulate organic matter (*S*_1_). The hydrolysis and acidogenesis/acetogenesis stages are considered together and the particulate organic matter is converted into VFA (*S*_2_) by the action of the hydrolytic microorganisms (*X*_1_):

Hydrolysis:

5 $$ k_{1} S_{1} \to X_{1} + k_{2} S_{1} + k_{4} {\text{CO}}_{2} + k_{1} k_{n} N $$

The methanogenic step involves the uptake of the VFA by the action of methanogenic microbes (*X*_2_) to produce methane:

Methanogenesis:

6 $$ k_{3} S_{1} \to X_{2} + k_{5} {\text{CO}}_{ 2} + k_{6} {\text{CH}}_{4} $$

The rate of methane production is directly related to the rate of the methanogenesis reaction by the coefficient k_6_ (20.29 L g^−1^) which has been modified from the original work to give the total methane flow rate in L day^−1^: the matrix description of the dynamic model is shown in Eqs. (7) and (8) and all the stoichiometric parameters in Eq. (5) and (6) can be found in Bernard et al. [5].

7 $$ \frac{{{\text{d}}\xi }}{{{\text{d}}t}} = Kr\left( \xi \right) + D\left( {\xi^{in} - \xi } \right) $$

8 $$ q_{\text{m}} = k_{6} r_{2} \left( \xi \right) $$

where

$$ K,r , \,$$$$ \xi $$ and $$ D $$ are expressed as shown in Eq. (9):

9 $$ \xi = \left[ {\begin{array}{*{20}c} {X_{1} } \\ {X_{2} } \\ {S_{1} } \\ {S_{2} } \\ C \\ N \\ Z \\ \end{array} } \right],\;r\left( \xi \right) = \left[ {\begin{array}{*{20}c} {r_{1} \left( \xi \right)} \\ {r_{2} \left( \xi \right)} \\ \end{array} } \right],\;K = \left[ {\begin{array}{*{20}c} 1 & 0 \\ 0 & 1 \\ { - k_{1} } & 0 \\ {k_{2} } & { - k_{3} } \\ {k_{4} } & {k_{5} } \\ {k_{n} k_{1} } & 0 \\ 0 & 0 \\ \end{array} } \right],\;D = I_{6} d $$

An important modification from the original model formulation is the inclusion of an additional dynamic state that represents the ammonia concentration in the digester (*N*). This was included to allow ammonia inhibition to be implemented the model screening process since this is an important phenomenon in AD of solid wastes [21]. The reaction stoichiometric coefficient for ammonia (*k*_n_) was calculated from the elemental composition of the waste multiplied by an estimated degradability coefficient (0.5 and 0.7 for GW and FW, respectively) and calculated as 1.033 and 1.842 mmol/g VS for GW and FW, respectively. Note that in the original model formulation, a pH variable was included and calculated as a function of the alkalinity, carbon and VFA state variables (Z, C and S_2_). However, this calculated variable had no impact on any other aspect of the model in terms of feedback inhibition and therefore this was omitted from this implementation.

#### Three reaction model (3R)

The 3R model includes a fractionation of the particulate organic matter into carbohydrates/fats (*S*_1a_) and proteins (*S*_1b_) and the hydrolysis stage consists of two reactions, namely hydrolysis of carbohydrate/lipid (10) and hydrolysis of protein (11). Each reaction produces VFA (*S*_2_) by the action of hydrolysis biomass (*X*_1a_ and *X*_1b_):

Hydrolysis of carbohydrates/fats:

10 $$ k_{1} S_{{ 1 {\text{a}}}} + k_{2} N \to X_{{ 1 {\text{a}}}} + K_{3} S_{2} + k_{4} {\text{CO}}_{ 2} $$

Hydrolysis of proteins:

11 $$ k_{5} S_{{ 1 {\text{b}}}} \to X_{{ 1 {\text{b}}}} + k_{6} S_{2} + k_{7} N + k_{8} {\text{CO}}_{ 2} $$

The methanogenic stage involves the conversion of VFA by the methanogenic population (*X*_2_) to methane as shown in Eq. (12):

Methanogenesis:

12 $$ k_{9} S_{2} + k_{10} N \to X_{2} + k_{11} CH_{4} + k_{12} {\text{CO}}_{ 2} $$

The methane flow rate is obtained using Eq. (13):

Methane flowrate

13 $$ q_{\text{m}} = k_{11} r_{2} $$

It should be noted that the Eqs. (10), (11), (12) and (13) have been adapted from [9]. As with the 2R model, Eq. (1) describes the general dynamics of the three reactions model with $$ K,r,  $$$$ \xi $$ and $$ D $$ expressed as shown in Eq. (14):

14 $$ \xi = \left[ {\begin{array}{*{20}c} {X_{{1{\text{a}}}} } \\ {X_{{ 1 {\text{b}}}} } \\ {X_{2} } \\ {S_{{ 1 {\text{a}}}} } \\ {S_{{ 1 {\text{b}}}} } \\ {S_{2} } \\ C \\ N \\ Z \\ \end{array} } \right],\;r\left( \xi \right) = \left[ {\begin{array}{*{20}c} {r_{{ 1 {\text{a}}}} \left( \xi \right)} \\ {r_{{ 1 {\text{b}}}} \left( \xi \right)} \\ {r_{2} \left( \xi \right)} \\ \end{array} } \right],\;K = \left[ {\begin{array}{*{20}c} 1 & 0 & 0 \\ 0 & 1 & 0 \\ 0 & 0 & 1 \\ { - k_{1} } & 0 & 0 \\ 0 & { - k_{5} } & 0 \\ {k_{3} } & {k_{6} } & { - k_{9} } \\ {k_{4} } & {k_{8} } & {k_{12} } \\ { - k_{2} } & {k_{7} } & {k_{10} } \\ 0 & 0 & 0 \\ \end{array} } \right],\;D = I_{7} d $$

Furthermore, the methane production coefficient for the 3R model (*k*_11_) has been modified to allow direct comparison with the experimental data of Sect. “Experimental method” (13.44 L g^−1^). For the stoichiometric parameters in Eqs. (10), (11) and (12), see Mairet et al. [9].

#### Kinetics of reaction

It has being reported in the literature that the first-order kinetic and Contois models are able to best describe the hydrolysis process [22], while the Monod kinetic equation has predominantly been used for soluble substrates with Haldane being frequently chosen to represent the methanogenesis reaction due to its sensitivity to VFA [5, 7]. In addition, the kinetic model for the growth of the microbial population by Tessier and Moser [14] is considered for the methanogenesis stage in the present investigation. Expression for biochemical conversion rates [2, 5, 7, 14] is shown in Eqs. 15–20 and the expressions for the ammonia and VFA inhibition factors (*I*_N_ and *I*_vfa_), applied by the multiplication by the rate of methanogenesis (*r*_2_), are shown in Eqs. 21–22 (modified from Batstone et al. [2]):

First-order:

15 $$ r = k_{\text{hyd}} S $$

Contois:

16 $$ r = \mu_{\hbox{max} } \frac{S}{{k_{s} + S}}X $$

Monod:

17 $$ r = \mu_{\hbox{max} } \frac{S}{{k_{s} + S}}X $$

Haldane:

18 $$ r = \mu_{\hbox{max} } \frac{S}{{k_{s} + S + \frac{{s^{2} }}{{k_{i} }}}}X $$

Moser:

19 $$ r = \mu_{\hbox{max} } \frac{{S^{\lambda } }}{{k_{s} + S^{\lambda } }}X $$

Tessier:

20 $$ r = \mu_{\hbox{max} } \left( {1 - e^{{ - \frac{S}{{k_{s} }}}} } \right)X $$

Ammonia inhibition:

21 $$ I_{N} = \frac{1}{{\frac{{k_{i,N} }}{N} + N}} $$

VFA inhibition:

22 $$ I_{\text{vfa}} = \frac{1}{{\frac{{k_{{i,{\text{vfa}}}} }}{{S_{2} }} + 1}} $$

#### Inorganic species

The non-organic compounds, including the inorganic carbon and nitrogen are included in the presented model. For a detailed description of the equilibrium expression for inorganic carbon, VFA and nitrogen, as well as the charge balance associated with the dissociation of the ions the reader should refer to Bernard et al. [5] and Mairet et al. [9]. Since the present work only considers the methane production rate for comparison with the experimental data the CO_2_ production and inorganic carbon state variable, as well as the alkalinity have not been reported since they have no mathematical influence on the methane production.

#### Model summary

In Sects. “One reaction model (1R), Two reaction model (2R), Three reaction model (3R), Kinetics of reaction, Inorganic species”, descriptions have been given for three AD model structures (1R, 2R, and 3R), a range of the kinetic rate equations that can be used to describe the reaction rates and two common inhibition functions. These model components can be combined to make a large number of different AD system models with varying complexity and ability to describe different phenomena. The ability of these models to reproduce the behaviour exhibited in the experimental results is tested to determine their suitability for modelling GW and FW digestion.

### Modelling methodology

The equations describing the dynamic variables of each model structure, the reaction kinetics and the inhibition function were implemented in Simulink (Mathworks, MA, USA) and solved numerically by employing a fourth-order Runga-Kutta method using the stiff solver ode15 s with a maximum step size of 0.002 days. Feeding pulses were represented as trapezoids in the dilution rate (*d*) with a duration of 0.004 days (~6 min) and height such that the integral of the flowrate for each pulse was equal to the volume of substrate added during each loading event as shown in Fig. 1.

The initial condition for the simulations was obtained by a simple parameter estimation performed on a batch incubation of the inoculum. In this method the sum of the concentration of particulate organic matter (*S*_1_) and hydrolytic (*X*_1_) and methanogenic organisms (*X*_2_) was assumed to be the measured VS of the sample (14.4 kg m^−3^). The methane production from the batch was then used to estimate the initial conditions and this method yielded the following conditions which were used in the semi-continuous simulations; *S*_1_ = 0.17 kg m^−3^, *X*_1_ = 7.75 kg m^−3^ and *X*_2_ = 6.48 kg m^−3^. The initial ammonia concentration of 75 mmol L^−1^ was based on a measured concentration of 1.28 gNH_3_ L^−1^ in the inoculum. As mentioned both in Sect. “Inorganic species”, C and Z had no impact on the model outputs of interest and were therefore not simulated. The descriptions of the green and food wastes are shown for each model in Table 1 including a justification for their selection.

**Table 1:** Feedstock description in the 1–3 reaction models (*β_1_ and β_2_ are part of the parameter estimation method)

State variable	Food waste $\left( {\xi_{in} } \right)$			Green waste $\left( {\xi_{in} } \right)$			Notes
Model	1R	2R	3R	1R	2R	3R	
*X*_1,1a,1b,2_	0	0	0	0	0	0	Assumed no X in substrate
*S*_1_	274	274	N/A	275	275	N/A	Based on measured VS
*S*_1a_	N/A	N/A	440β_1_	N/A	N/A	392β_1_	Based on measured VS and COD_th_
*S*_1b_	N/A	N/A	440β_2_	N/A	N/A	392β_2_	Based on measured VS and COD_th_
*S*_2_	N/A	197.7	12.65	N/A	72.1	4.61	Based on measured VFA
N	N/A	0	0	N/A	0	0	Assumed no NH_3_ in substrate

### Parameter estimation and parameter uncertainty

The parameter estimation technique used the non-linear least square method as supplied with the optimisation toolbox in Matlab (Mathworks, MA, USA). A multi-start strategy was employed where several different initial parameter sets were used to avoid the minimisation algorithm reaching a local minimum [23]. Despite using simplified models, in all cases investigated, except the 1R model, the number of parameters prohibits the estimation of a full parameter set. Therefore, the focus of this paper has been on identifying and assessing the suitability of a model by varying the parameters describing the reaction kinetics and inhibition rather than stoichiometry.

The exclusion of stoichiometric parameters (*k*_*n*,_*β*) from the estimation method can be justified since they should not significantly impact on the nature of the feedstock or process conditions. The exception to this is parameter(s) that expresses the yield of VFA from the degradation of the feedstock (*k*_1_ in the 2R model and *β*_1_ and *β*_2_ in the 3R model) since, for solid wastes, this can be highly variable due to two main factors; the concentration of non-biodegradable substances including water, and the biochemical makeup of the organic material (e.g. lignin, fats, carbohydrates, etc.). Therefore, in the present investigation these parameters were critical to allowing a good fit of the model. Further, it should be noted that previous authors did include these parameters in their identification procedure and therefore this could be seen as a shortfall of these works [1, 6, 8].

In summary, the parameters that were estimated were the biomass to VFA stoichiometric parameters (*k*_1_, *β*_1_, *β*_2_), the kinetic parameters (*k*_hyd_, *k*_x_, *k*_s_, *µ*_max_, *λ*) and the inhibition parameters (*k*_i_, *k*_i,vfa_, *k*_i,N_). This means that the parameters estimated for each model combination varied between 2 in the simplest case (1R with the first-order kinetics and no inhibition) and 11 in the most complex model (3R with Contois hydrolysis, Moser methanogenesis, and VFA and ammonia inhibition).

Parameter sets for the mechanistic model of AD systems are not generic and are developed for specific cases, makes the estimation of its parameters specific for the case under examination. The standard for the decision on which the model best describes the physical phenomena involves finding the optimal solution of the model parameters based on a cost function. In this case, the cost function is given by Eq. (23), this is simply the sum of the square between the model and the experimental data points, and it is commonly used for parameter estimation studies in this field [13, 23, 24]. Nevertheless, to measure the extent of the deviation of the model results from observed value obtained from experimental investigations, the relative root mean square error (rRMSE) is implemented since this allows comparison of the data obtained from different experiments and it is expressed as a percentage of the time-based mean of the measured methane flow rate (*σ*_qm,exp_).

It should be noted that only the measurements for methane flowrate are used for parameter estimation, rather than including other offline measurements, e.g. VFA. This choice was made because the flowmeter provided continuous online measurement and therefore many thousands of data points for use in parameter estimation whereas offline data only provided a small number of data.

23 $$ j = \hbox{min} \sum\limits_{i = 1}^{n} {\left( {q_{\text{m,exp}} - q_{\text{m}} } \right)^{2} } $$

24 $$ {\text{rRMSE}}\,\left ( \% \right) = 100\frac{{\sqrt {\left( {\frac{j}{n}} \right)} }}{{\sigma_{{q_{{{\text{m}},\exp }} }} }} $$

The standard errors associated with the parameter estimation technique were calculated as the diagonal elements of the square root of the inverse of the Hessian matrix with respect to the cost function (Eq. 23).

## Results and discussion

### Experimental results

The two laboratory digesters were fed the equivalent OLR, on a COD basis, of GW and FW, respectively, which were characterised as presented in Table 2 including the calculation of COD_th_. Despite the same OLR, the behaviour of the digesters, both in terms of the methane production rate and the mode of failure, was strikingly different due to the different compositions and degradability of the organic wastes. For the GW and FW fed systems, respectively, the average methane production over the course of the experiment was 0.67 and 2.38 L day^−1^ and the specific methane production was 0.114 and 0.233 L g^−1^ COD_added_ (0.176 and 0.404 L g^−1^ VS_added_).

**Table 2:** Measured feedstock characteristics

Characteristic	Unit	GW	FW
TS	g L^−1^	402	301
VS	g L^−1^	275	274
Ash	% of TS	34.88	10.27
C	% of TS	34.66	49.15
H	% of TS	4.50	7.56
N	% of TS	1.98	3.35
S	% of TS	0.03	0.03
O	% of TS	23.95	29.64
COD_th_	g COD g^−1^ VS	1.55	1.73
VFA	g COD L^−1^	4.61	12.65

The aim of the experiment was to produce rich kinetic data of the methane production rate and eventually a failure of the system due to organic overload. However, in the case of GW, the system failed due to excessive foaming before there were any signs of organic stress (increased VFA, reduced specific methane production), at about day 110 and a maximum OLR of 5.52 g COD L^−1^ day^−1^ (experimental average 2.90 g COD L^−1^ day^−1^). For the FW system the organic failure of the system was observed with an increase in the VFA concentration to 18 g COD L^−1^ at day 160 and a reduction in the methane production despite continued, albeit reduced, organic loadings. The maximum and experimental OLR in this case were 15.03 and 5.15 g COD L^−1^ day^−1^, respectively, and the experiment was terminated after 175 days.

**Fig. 2:**
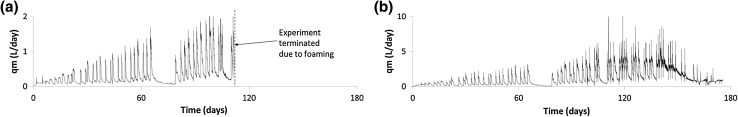
Experimental methane production for the digestion of **a** GW and **b** FW

The methane production rate from the two systems is shown in Fig. 2 and these data form the basis, and the sole input, for the parameter estimation and assessment the of model suitability. The number of data points was 4073 and 23644 for GW and FW, respectively, and it should be noted that although data were collected by intermittent sampling for VFA, TS and VS, these did not form input into the parameter estimation method.

### Suitability assessment of model structures, reaction kinetics and inhibition models

**Fig. 3:**
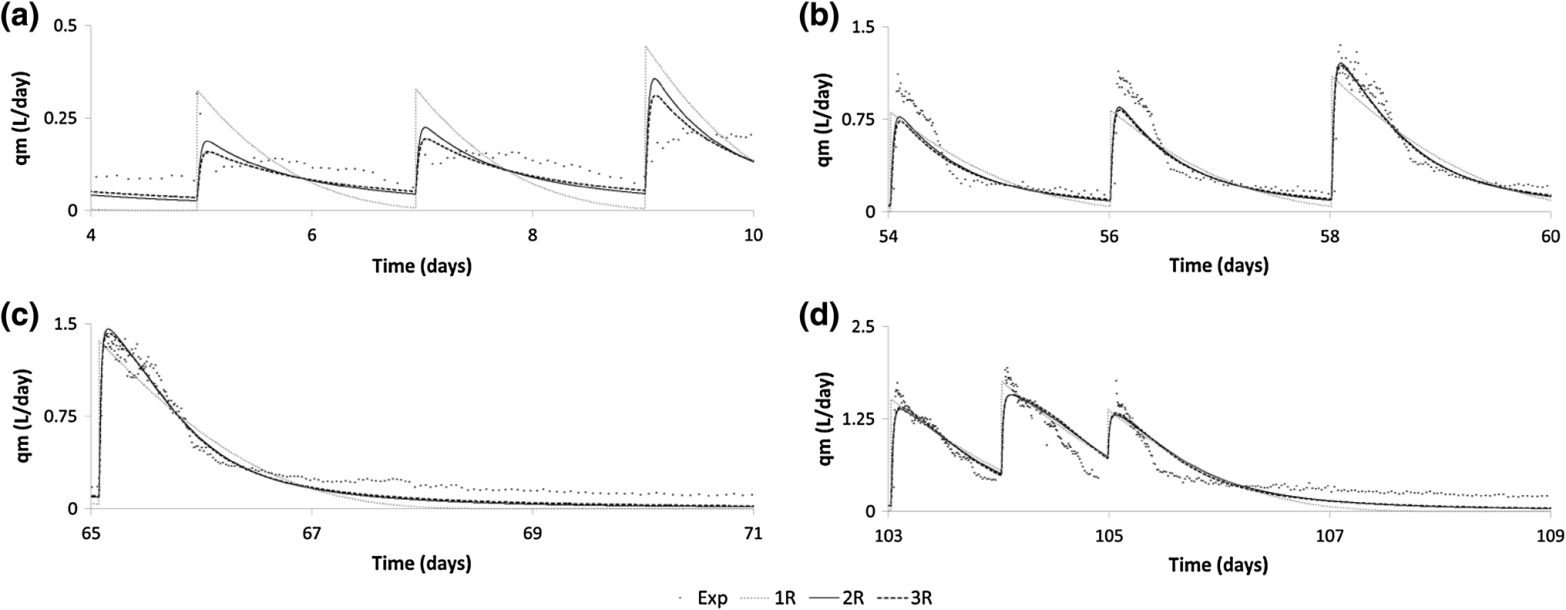
Methane flowrate for digestion of green waste showing the best fitting model combinations (1R, 2R, 3R) and experimental data for periods **a** 4–10, **b** 54–60, **c** 65–71 and **d** 103–109 days

**Fig. 4:**
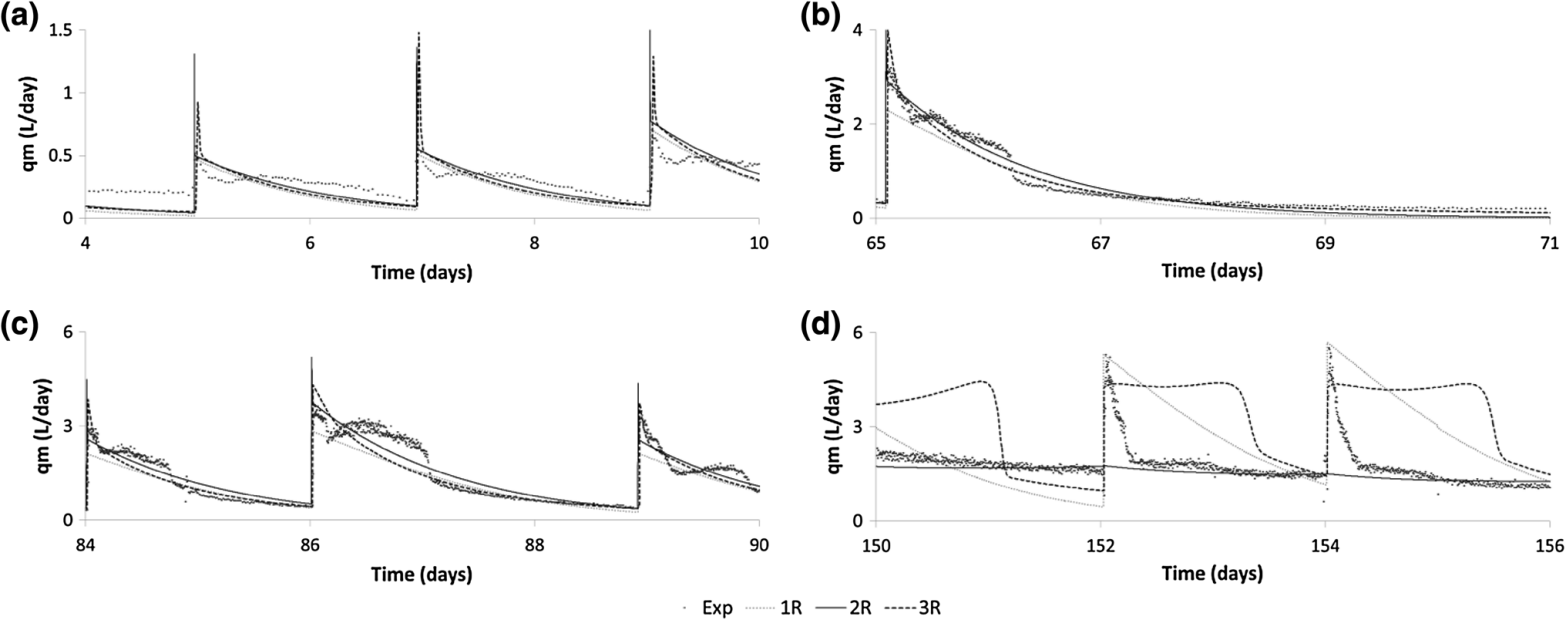
Methane flowrate for digestion of food waste showing the best fitting model combinations (1R, 2R, 3R) and experimental data for periods **a** 4–10, **b** 65–71, **c** 84–90 and **d** 150–156 days

The assessment criteria for the suitability of a model to represent the experimental data were the minimum rRMSE between the experimental data and the model with the best fitting parameter set as found by the parameter estimation method. For each broad model structure (1R, 2R, 3R), different reaction kinetics are shown in the Sect. “Kinetics of reaction” and these were tested along with VFA and ammonia inhibition in the cases of the 2R and 3R models. The results of the 2R parameter estimation for each of the kinetic combinations are shown in Tables 3 and 4 for GW and FW, respectively, and the best fit parameters for each model structure is shown in Table 5. The simulated methane production predicted by best fitting case of each model is plotted against excerpts of the experimental data in Figs. 3 and 4 for GW and FW, respectively.

**Table 3:** rRMSE (%) between experimental and model data for the 3R model with combinations of reaction kinetics and inhibition for the AD of GW (*Model chosen as most suitable)

Inhibition	Methanogenesis		Monod	Haldane	Moser	Tessier
None	Hydrolysis	First order	22.6	NA	21.9*	22.5
		Contois	22.6	NA	21.9	22.7
		Monod	22.6	NA	21.9	22.5
NH_3_		First order	22.6	NA	22.0	22.5
		Contois	22.6	NA	22.0	22.5
		Monod	22.5	NA	21.9	22.4
VFA		First order	22.5	22.2	26.8	22.5
		Contois	22.6	22.5	23.3	22.5
		Monod	22.5	22.2	21.9	22.4
VFA & NH_3_		First order	22.5	22.3	21.9	22.5
		Contois	25.8	22.6	23.6	22.5
		Monod	22.5	22.3	21.9	22.5

**Table 4:** rRMSE (%) between experimental and model data for the 2R model with combinations of reaction kinetics and inhibition for the AD of FW (*Model chosen as most suitable)

Inhibition	Methanogenesis		Monod	Haldane	Moser	Tessier
None	Hydrolysis	First order	34.9	NA	34.6	34.8
		Contois	35.2	NA	37.3	35.3
		Monod	38.1	NA	34.6	34.8
NH_3_		First order	37.0	NA	33.6	35.2
		Contois	33.7	NA	36.3	33.9
		Monod	36.9	NA	33.6	35.0
VFA		First order	61.8	34.9	36.0	37.9
		Contois	35.2	29.5	30.4	29.0
		Monod	33.0	33.9	39.1	38.4
VFA and NH_3_		First order	72.3	64.3	37.3	31.8
		Contois	27.9	27.2*	27.3	38.8
		Monod	28.2	28.1	32.1	37.7

**Table 5:**
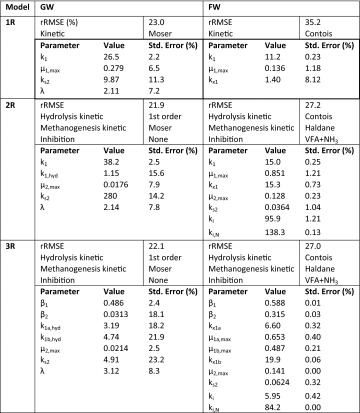
Parameter values for GW and FW digestion for the best fitting models with 1R, 2R and 3R structures

#### 1R model

Results obtained from the 1R model parameter estimation reveals that the Moser kinetic equation was most suitable for describing the GW methane production with an rRMSE of 22.9 %. Tessier, Contois, Monod and first-order kinetic equation gave an rRMSE of 23.5, 23.6, 23.6 and 25.1 %, respectively. For FW the best fit was the Contois kinetic equation with an rRMSE of 35.3 %, whereas the rRMSE for the Moser, Tessier, Monod and first-order were 37.6, 38.1, 39.6 and 39.7 %, respectively. It is not easy to draw a strong conclusion from this since the results are not strongly dependent on the choice of the kinetic equation. 

#### 2R model

When the model complexity was increased by the addition of another reaction (2R) it was found that in the case of GW there was a slight reduction in the minimum rRMSE to 21.9 % when using the combination of first-order/Moser kinetic for Hydrolysis and Methanogenesis, respectively. Again the results of the parameter estimation procedure showed low sensitivity to the choice of the reaction kinetics suggesting that all of the kinetic rate equations could describe equally well the phenomena exhibited in the GW experimental data, as shown in Table 3. Furthermore no significant improvement in model fitting was found by the introduction of two common forms of inhibition in AD systems, namely VFA and ammonia. We can use this to deduce that it was unlikely that inhibition by either species was affecting the kinetics of biomethane production, at least by a mechanism that could be replicated by the Eqs. (21) and (22). This hypothesis can be supported, in the case of ammonia inhibition, by the low nitrogen content measured in the feedstock, and the low biodegradability measured in the methane production data which together mean that limiting ammonia conditions were unlikely in the GW fed system. In the case of VFA inhibition, the measured VFA concentration in the effluent from the GW system never reached more than 0.1 g COD L^−1^.

The ability to describe the fermentation of ethanol by employing the Moser kinetics has been reported in the literature [25]. In the case of GW it was found that all of the best fitting model combination used the Moser kinetic equation for the methanogenic reaction. Of the kinetic combinations producing the lowest rRMSE (21.9 %) the first-order/Moser combination was chosen for further analysis since it is the simplest, as the first-order kinetic has only a single parameter, and additionally that first-order has been traditionally used for the description of hydrolysis organic matter [2] and this has been validated experimentally [26] as well as for surface related processes [22].

When assessing the suitability of the 2R model to reproduce the FW methane production data, it was found that the minimised rRMSE was greatly reduced compared with the 1R model, to 27.2 % when both ammonia and VFA inhibition were included and the Contois/Haldane combination was used. The selection of Contois as the best performing hydrolysis can be attributed to the fact that it allows the hydrolysis rate to be controlled by both the substrate and microorganism concentration, i.e. both the mass transfer limitation governed by available surface area, and the growth limited condition during periods of high feeding rates or changes in OLR [27]. This is especially relevant since there are large changes in OLR in the FW experiment which could have caused the first-order model for hydrolysis to be deficient. The use of the Contois equation for the representation of hydrolysis stage of AD has been extensively reported in the literature [28–30] which agree with our findings. Further, the Haldane type kinetic model has been used extensively for modelling the methanogenic stage of anaerobic digestion process, since it incorporates the effects of inhibition by VFA [2, 9].

Standard errors associated with the estimated parameters were increased compared with the 1R model, in the case of GW, to a maximum of 15.6 % (c.f. 11.3 % for 1R) whereas the errors for FW remained low with a maximum of 1.21 %. For GW, the increased uncertainty in the parameters estimated in this way could indicate several related issues; that the dataset is not sufficiently rich such that the parameter values can be confidently estimated, or that the number of parameters estimated and/or model complexity leads to no distinct solution in the case where parameters are co-correlated with the output data (over-parameterised). For FW, there are several factors which can explain the low levels of uncertainty associated with the parameter values: First the dataset is larger than for GW both in terms of length of the experiment and in terms of number of gas flow data points, since the biogas production from FW was higher; second the degradation kinetics are more complex. This is the case both in terms of the characteristic shape of the methane flowrate after feeding which indicates some temporary inhibition of the methane production, and also in the period of severe inhibition during the organic failure of the system. Together these factors meant the dataset was more rich in information, especially regarding these additional phenomena, which in turn ensured that errors associated with the parameters remained low while the ability of the more complex model to reproduce the experimental data increases as shown by the reduced rRMSE.

#### 3R model

To avoid an exhaustive screening procedure, the application of the 3R model was limited to the best fitting kinetic combinations and inhibition models, as found in the 2R model study. In the case of GW, there was a minor reduction in the quality of fit compared with the 2R model (rRMSE = 22.1 % c.f. 21.9 %) and observation of the best fit parameters shown in Table 5 show that the parameter estimation algorithm found an optimum solution using only one of the two substrate fractions. This is demonstrated by the low value of β_2_ compared with β_1_, meaning that the degradation of the predicted protein fraction had very little influence on the simulated methane production. This can also be seen in Fig. 3 where the predictions of the 2R and 3R model are almost identical showing that, using the model structures provided, the characteristic kinetic of methane production cannot be better represented by two particulate fractions degrading with differing kinetic behaviours. This is in contrast to the results of Batstone et al. [31]. As in the case of the 2R parameter estimation, the standard errors associated with the 3R GW case are rather high (maximum 23.2 %) indicating that the model is somewhat over-parameterised given the richness of the dataset. In this case the increased uncertainty combined with no improvement in goodness of fit indicates that the 2R or 1R model should be recommended.

In contrast to the results for GW, there was a slight improvement of the fit when comparing 3R with 2R (rRMSE = 27.0 % c.f. 27.2 %) for the FW data, and additionally the improvement was associated with the prediction of two distinct particulate fractions as shown in the values of *β*_1_ and *β*_2_ (0.588 and 0.315). The effects of this particulate fractionation can be seen in the methane Fig. 4b, c where the methane flow predicted by the 3R model shows slightly improved fit of the complex kinetic behaviour shown in the experimental data shortly after each feeding. This can be related back to the Contois kinetic degradation of the two fractions which have differing saturation constants. Note that the standard errors associated with the parameters using the FW data remain low, with a maximum of 0.4 %, owing to the richness of the dataset as previously discussed and the use of the 3R, along with the 2R model can be recommended above the 1R model.

### Model descriptions and qualitative fit

A detailed, but qualitative, examination of the fit between the different models and experimental data allows assessment of the phenomena that each model is able to reproduce and therefore some recommendations may be made. Along with the full description of the methodology used, the results shown in Table 5 and the discussion below will allow other researchers to make an informed assessment of whether the presented parameter values suit the needs of future modelling work.

#### Start-up

All of the models investigated show a poor fit with the experimental data at the start of the experiment, namely during the period 0–15 days, as shown in Figs. 3a and 4a. This is likely to be due to the inoculum being disturbed during its collection, transport and processing in the laboratory and also being poorly acclimatised to the chemical makeup of the new substrate (FW or GW) since the inoculum was originally sourced from a sewage sludge digester. Recent studies have argued the need for adequate monitoring and analysis of the microbial diversity for the purpose of gaining a better understanding of the complexity of the AD process since the current methods of analysis are lacking and/or are specific to a particular set of microorganisms [9, 32]. Generally, anaerobic microorganisms, especially methanogens, require a stable temperature for their continued effectiveness and a disruption of this state destabilises their overall activity in a new environment. Further, the contamination of the process by oxygen ingress during processing could also contribute to the poor model fit with the experimental data, particularly at the beginning of the experiment, and perhaps more importantly, during the development of a rebalancing of the microorganism populations caused by the new substrate composition [33]. The phenomena of temperature dependence, oxygen stress and population acclimatisation are not modelled and therefore these complex behaviours cannot be captured, and therefore, the use of these models is not recommended for the simulation in the initial start-up phase of an AD system.

#### Green waste model fit

The model fitting during the remaining phase of the GW experiments (Fig. 3b–d) is qualitatively better than at the start of the experiment presumably because the experimental system was not experiencing the population shifts associated with acclimatisation and also not under stress for organic overload or inhibition. The differences between the 1-3R model predictions are relatively small, but it can be observed that the 2R and 3R model tend to fit slightly better in two aspects; first in the initial build-up of methane production after a feeding event, and second in the subsequent decay in methane production. The former is true because the structure of the 2R and 3R models allows the delay in methane production due to the formation of the intermediate volatile fatty acid species, whereas the 1R model instantly shows methane production based on the current particulate substrate concentration. The differences in the decay in methane production can be seen most clearly in the period of no feeding between 65 and 71 days where 2R and 3R models show a more sustained methane production. In the physical system, this phenomenon has two components; (1) the substrate contains a very slowly degradable fraction which continues to release soluble matter over long periods of time and thus contributing to a long term, albeit low, production rate of methane, and, (2) the death of microorganisms gives the living population a continuous (but dwindling) supply of fresh substrate. Whilst the first of these could be captured by the 3R model the estimation method has not identified this as an optimal solution for GW as shown by the very low value of β_2_. The latter of these phenomena cannot be captured by the 1-3R models as formulated in this work whereas this is included in ADM1 where the decay of the microorganism populations is recycled back to form new degradable organic matter.

Since the methane flow data for the GW experiment did not contain information relating to an organic overload event (in contrast to the FW experiment), the use of these models/parameters to predict the behaviour of a system in these conditions is not recommended. However, it is clear that the predominant failure mode for the GW digester was foaming, and the 1-3R GW models continue to fit well to the experimental data until the repeated foaming events caused the experiment to be terminated. This shows the inadequacy of the simplified models to predict complex phenomena outside of their scope.

#### Food waste model fit

The modelled methane flow rate during the ‘acclimatised’ period for the FW experiments is shown in Fig. 4b, c, d. This shows distinct qualitative differences between the 1-3R models in their quality of fit to the experimental data, thus agreeing with the quantitative assessments described in Sect. “Suitability assessment of model structures, reaction kinetics and inhibition models”. The 3R and 2R models appear to capture the organic overload condition during the latter parts of the experiment (Fig. 4d), which corresponds to the accumulation of VFA and inhibition by ammonia. However, the distinction between the 3R and 2R models was that the 3R model was better able to capture the characteristic shape in the degradation kinetics for the period following a feeding event and even during the long period without feeding during the days 65–71. This is because the parameter estimation algorithm identified a solution that described the FW with a two distinct particulate fractions that behaved differently, due to their saturation constants, directly following a feeding event, leading to better fit of the initial methane flow peak, and additionally in the subsequent decay in methane production. Clearly, this is closer to reality than the single input fractions used for the 1R and 2R models since both show characteristic exponential decay curves in the methane production rate after a feeding event which does not follow the experimental data.

#### Model validation

**Fig. 5:**
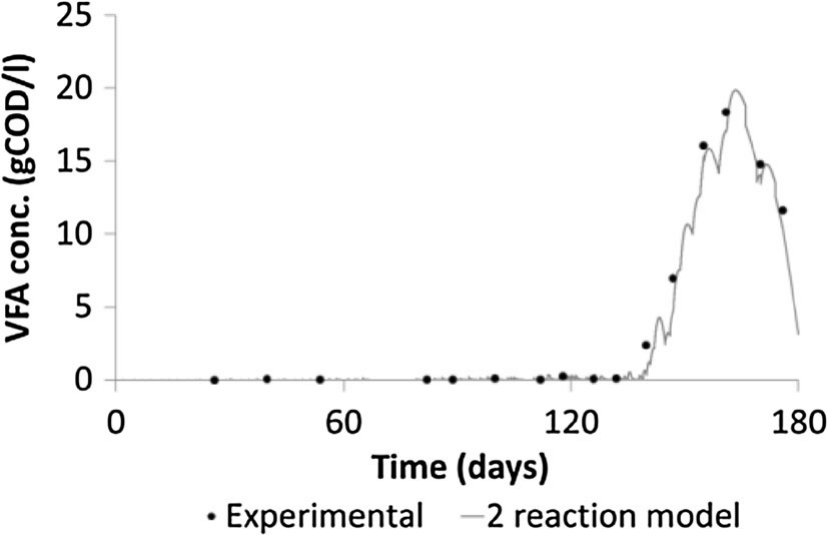
Model and experimental VFA data for AD of FW with 2R model

For model validation the goodness of fit between the experimental data and the model output was evaluated by the calculation of the coefficient of determination. This was calculated using the same experimental datasets of the methane flow used for the parameter estimation. In the case of green waste (GW), the model showed a strong correlation for all three models; *r*^2^ = 0.91, 0.92 and 0.89 for 1R, 2R and 3R, respectively. While for food waste FW; *r*^2^ = 0.70, 0.80 and 0.70 for 1R, 2R and 3R, respectively. While the 3R model captured some key phenomena for the degradation of FW and showed a lower rRMSE, it did not show such a strong correlation when checked against the experimental data. Additionally it is interesting to note that the 2R model predicted well the concentration of the VFA in the system in Fig. 5. This shows a good agreement in both the rise and fall in the VFA (S2) and this gives some validation to the parameter set found in the 2R model for FW under organic/ammonia stress since the VFA data did not form part of the estimation method and its closeness of fit is purely down to the mechanistic nature of the model and the parameters estimated from methane production.

#### Model fit summary

The models presented in the study have shown the ability to represent some of the major phenomenon in AD, albeit to variable degrees, and hence may be suitable for some modelling applications depending on the objectives. For GW the 1R or 2R models are more suitable when considering the quality of fit and parameter uncertainty, and inclusion of inhibition by ammonia or VFA shows no improvement. For the FW a more complex 2R or 3R model is needed along with VFA and ammonia inhibition. These main results can be related back to both the characterisation work that was presented in Table 2 and the known degradation characteristics of the two biomass feedstock samples used. GW contains a high fraction of non-degradable organic matter in the form of lignocellulose and a relatively low nitrogen content which combined with the low degradability leads to reduced ammonia release upon degradation compared with other feedstocks. This means that neither VFA inhibition, associated with organic overload conditions, nor ammonia inhibition, associated with elevated ammonia concentrations, should be important phenomena in the degradation of GW in AD under normal operating conditions, which is in agreement with the results of this study. On the other hand FW is more degradable, contains a mixture of both rapidly (e.g. sugars, fatty acids) and slowly degradable components (e.g. cellulose, hemicellulose) and a higher degradable nitrogenous fraction which leads to higher concentrations of ammonia upon degradation. The combined result is that the degradation kinetics are more complex and that inhibition by both VFA and ammonia are important. Again the physical model agrees with the modelling outcomes of this study. However, the characteristics of degradation of the feedstock cannot be predicted from the feedstock analysis given in Table 2 alone since these only give some physical and chemical analysis and no information is presented here regarding the overall degradability and the associated rate of degradation, which both have a large impact on the AD process.

The applications of these models could be for online monitoring and control of AD processes due to the vastly reduced computational cost and effort relative to large complex models [5, 9] as well as the ease of recalibrating the dynamic state variables in real time. The models are flexible in that new state variables can easily be introduced based on the objectives of the modeller, e.g. if long-term methane production (between feeding events) is of interest then a microorganism decay mechanism could be added. The limitations of these models have been elucidated here and they need to be understood before their application.

### Sensitivity analysis

**Fig. 6:**
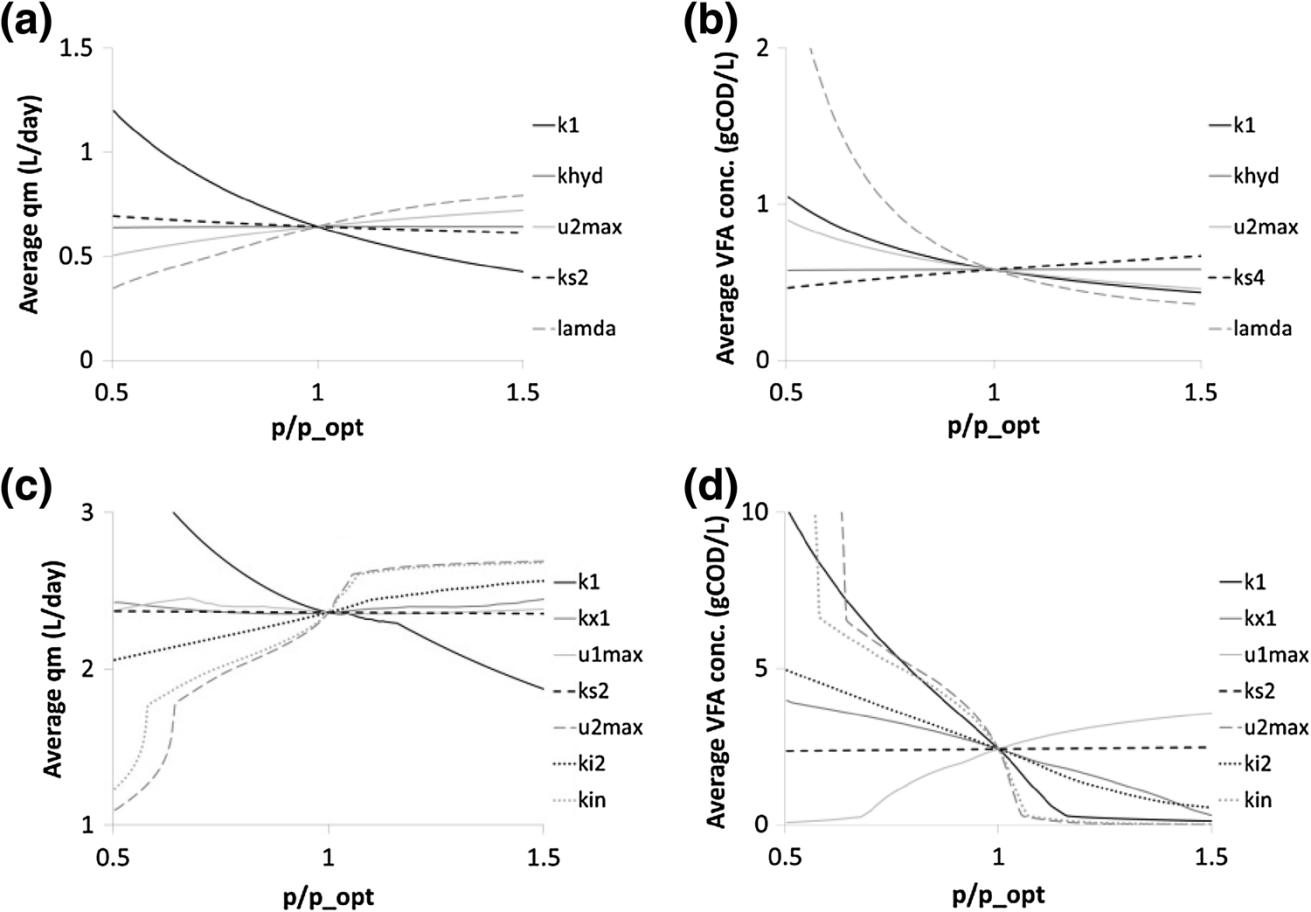
Local sensitivity analysis of the best fit parameters set (p_opt ±50 %) for the simulation results of the average methane flow (*q*_m_) and VFA concentration (*S*_2_) over the whole experimental period for (**a**, **b**) GW and (**c**, **d**) FW

For AD systems, the sensitivity analysis is local in nature and it is usually presented as the variation in the output signal with respect to the parameters [24]. In fact the analysis performed by Bernard et al. [5] showed that the kinetic parameters (*k*_s_ and *µ*_max_) stoichiometric yield coefficients (*k*_1_, *k*_2_, *k*_3_, *k*_6_), and the Inhibition constant (*k*_i_) were the most important parameters in terms of methane production sensitivity. In fact this list was used to choose the parameters for estimation in this study, neglecting the stoichiometric yields beyond *k*_1_ (*k*_2–6_) as these could be considered fixed. For the purpose of this work a local sensitivity analysis was performed by exploring the parameter space surrounding the ‘optimum’ parameter set as located by the parameter estimation method (p_opt_), thus giving some insight into the relative importance of each parameter at the chosen operating point. Figure 6 shows the results obtained from the best fitting 2R models, for both FW and GW, with the sensitivity being expressed as the average of methane flowrate (*q*_m_) and VFA concentration (*S*_2_) over the experimental period. In the case of GW it was found that the degradation factor (*k*_1_), the maximum uptake rate of VFA (*µ*_2,max_) and the index of the substrate concentration (*λ*) were the most influential parameters, while the solution was much less sensitive to the first-order coefficient (*k*_hyd_) and the half saturation (*k*_s_). Similarly, for food waste, the parameters with the most significant influence on the solution were the ammonia inhibition constant (*k*_i,N_), the VFA inhibition constant (*k*_i_) and the uptake rate of VFA (*µ*_2,max_). The less sensitive parameters include the degradation factor, the uptake rate of the hydrolysis stage (*µ*_1,max_), the Contois half saturation constant (*k*_x_) and the half saturation constant for the VFA degradation (*k*_s_).

Further, to verify the results of the local sensitivity analysis, a global sensitivity analysis was performed, focusing only on the estimated parameters, using a Monte-Carlo method with the variation in each parameter being ±50 % with a uniform probability distribution and 2000 sampling points. The results obtained are shown in Table 6 and are represented by correlation coefficients between the average methane flow and each parameter. Upon inspection, the analysis gives a similar outcome to the local analysis in terms of the relative sensitivity of the average methane flow rate to the parameter variations.

**Table 6:** Global Sensitivity analysis correlation coefficients (*r* ^2^) between parameter values and average methane flowrate for the best fitting two reaction models for FW and GW

GW		FW	
Parameter	*r* ^2^	Parameter	*r* ^2^
*k*_1_	−0.76	*k*_1_	−0.40
*k*_1,hyd_	0.03	*µ*_1,max_	0.04
*µ*_2,max_	0.30	*k*_x1_	−0.08
*k*_s2_	−0.15	*µ*_2,max_	0.60
*λ*	0.49	*k*_s2_	0.04
		*k*_i,vfa_	0.14
		*k*_i,N_	0.47

It is worth emphasising that in this paper, the model parameter(s) representing the overall stoichiometry of the first reaction step (*k*_1_ in 1R and 2R models, *β*_1_ and *β*_2_ in 3R model) was included in the parameter estimation method, and this is in contrast with some other similar work. This can be easily justified by the outcome of the sensitivity analyses, which shows that the model outputs have a high dependence on these parameters. Further to this, these parameters are largely dependent on the characteristics of the feedstock being digested since they must describe both the moisture content as well as the fraction of the organic material that is degradable. This implies that they should be considered, along with the kinetic parameters, to be feedstock specific.

### Conclusion

The main results reveal that AD models containing up to three biochemical reactions are able to fit experimental methane production from solid waste samples of both GW and FW with a minimum rRMSE of 22 and 27 % over experimental periods of 112 and 176 days, respectively. It was observed that the model structure, both in terms of the number of reactions, and inhibition, plays a key role in the ability to accurately describe the experimental data, rather than the choice of kinetic equation to determine the reaction rate. In the case of GW, the results showed that either a one or two reaction model could fit the experimental data with no improvements from the addition of a third reaction or inhibition effects. The situation with FW was more complex and increasing the number of reactions, as well as the inclusion of inhibition by VFA and ammonia improved the quality of fit. The two reaction model was able to reproduce the elevated levels of VFA during a period of organic overloading.

## Appendix: nomenclature

**Table:**

Symbol	Meaning	Unit in 2R model	Unit in 3R model
*C*	Concentration of inorganic carbon	mmol L^−1^	mmol L^−1^
*d*	Dilution rate	Day^−1^	Day^−1^
*I*_N_	Ammonia inhibition rate factor	None	None
*I*_vfa_	VFA inhibition rate factor	None	None
*k*_n_	Reaction stoichiometric coefficient n	Various	Various
*k*_x_	Contois half saturation constant	g COD g^−1^	g COD g COD^−1^
*k*_s_	Half saturation constant	mmol L^−1^	g COD L^−1^
*k*_i_	Haldane inhibition constant for VFA	mmol L^−1^	g COD L^−1^
*k*_i,vfa_	General inhibition constant for VFA	mmol L^−1^	g COD L^−1^
*k*_i,N_	General inhibition constant for ammonia	mmol L^−1^	mmol/L
*n*	Number of experimental data points	# of points	# of points
*N*	Concentration of ammonia	mmol L^−1^	mmol L^−1^
*p*_opt_	Best fitting parameter set	Various	Various
*q*_m_	Modelled methane flowrate	L day^−1^	L day^−1^
*q*_m,exp_	Experimental methane flowrate	L day^−1^	L day^−1^
*S*_1_	Concentration of organic substrate	g VS	N/A
*S*_1a_	Carbohydrate and fats concentration in substrate	N/A	g COD L^−1^
*S*_1b_	Protein concentration in substrate	N/A	g COD L^−1^
*S*_2_	Concentration of VFA in two reaction model	mmol L^−1^	g COD L^−1^
*X*_1,1a,1b_	Concentration of hydrolysis biomass	g L^−1^	g COD L^−1^
*X*_2_	Concentration of methanogenic biomass	g L^−1^	g COD L^−1^
*Z*	Total alkalinity	mmol L^−1^	mmol L^−1^
*ξ*	Vector of state variables	Various	Various
*µ*_n_	Specific growth of microorganism n	Day^−1^	Day^−1^
*µ*_n,max_	Maximum growth rate of microorganism n	Day^−1^	Day^−1^
*σ*_qm,exp_	Mean experimental methane flowrate	L day^−1^	L day^−1^
